# Monitoring Pig Structural Soundness and Body Weight in Pork Production Systems Using Computer Vision Approaches

**DOI:** 10.3390/ani15050635

**Published:** 2025-02-21

**Authors:** Ryan Jeon, Caleb Rykaczewski, Thomas Williams, William Harrington, James E. Kinder, Mark Trotter

**Affiliations:** 1 Integer Technologies LLC, 1556 Main Street, Suite 200, Columbia, SC 29201, USA; 2 Department of Animal Sciences, College of Food, Agricultural, and Environmental Sciences, The Ohio State University, Columbus, OH 43210, USA; rykaczewski.2@buckeyemail.osu.edu (C.R.); kinder.15@osu.edu (J.E.K.); 3 Institute of Future Farming Systems, School of Health, Medical, and Applied Sciences, CQUniversity, Rockhampton, QLD 4701, Australia; t.m.williams@cqu.edu.au (T.W.); m.trotter@cqu.edu.au (M.T.); 4 College of Business, Law, and Governance, James Cook University, Townsville QLD 4811, Australia; william.harrington@my.jcu.edu.au

**Keywords:** precision technologies, computer vision, pork production, weight, skeletal structure

## Abstract

As the global demand for products from food-producing animals increases with greater household economic capacity, there is an increased emphasis on the development of precision technologies for monitoring the health, product production, and wellbeing of these animals. The present review focuses on pork production. Using these systems is advantageous for enhancing pork production efficiency when trained personnel utilize these technologies to full capacity and have objective, automated, and uninterrupted streams of data collection. While these systems have great potential for revolutionizing food animal production, the nascent stage of computer vision in precision technology has precluded its integration into traditional agricultural practices and systems. In this review paper, there is a focus on the need to (1) evaluate the performance and effective use of computer vision technologies to collect and evaluate reliable data from pork production enterprises; and (2) focus on the current state of sensor-based animal management using a data fusion approach to monitor pig health/performance. Many of these technologies are in various stages of development; therefore, these technologies have not been integrated into pork production or other food animal producing systems. Even though the focus of this review article is on the utilization of these technologies in pork production systems, these technologies are relevant in other food animal production systems, particularly dairy and poultry production. Therefore, we describe an approach that emphasizes the important need for computational capacity and speed, edge computing, data storage and transmission, and maintaining connectivity in rural settings.

## Simple Summary

Opportunities exist to integrate computer vision systems into pork production to enhance monitoring and decision-making, particularly in the areas of structural soundness, lameness, and body weight prediction. These aspects are important for maintaining herd health, optimizing productivity, and ensuring economic viability. Traditional methods for assessing pig body weight and leg structural soundness are labor-intensive, subjective, and often inaccurate. Computer vision offers an opportunity by providing automated, noninvasive, and precise assessments, making these tasks more efficient and reliable. With the capability to autonomously monitor important physical traits using deep learning models and 3D imaging techniques, computer vision systems are likely to be important in the future of pork production. In this review, we explore the current advancements, challenges, and future potential of computer vision in pork production systems.

## 1. Conventional Approaches to Visual Assessment of Pigs in Breeding Herds

The focus of this review article is on body weight and lameness. These factors have a large effect on the cost of pork production [1] and are aspects where initial progress can be made using precision technologies. Feet and leg structural soundness and body weight are important pig characteristics that require regular monitoring and evaluation to ensure the economic viability of pork production systems; however, accurately assessing these features is difficult. The assessment of feet and leg skeletal characteristics associated with structural soundness is traditionally conducted by those trained in making these visual appraisals. In an industry with a relatively large workforce turnover, this reliance can lead to inconsistencies and inaccuracies in these evaluations [2]. Subjective visual evaluations, though requiring minimal time to conduct, lack consistency and accuracy [1]. Similarly, human visual appraisal of pigs for marketing estimation leads to inaccuracies [3].

### 1.1. Foot and Leg Structure in Sows

In pork production systems, structural soundness of the feet and legs is a major problem. Depictions of typical leg structural problems can be accessed via this link: https://i0.wp.com/porkgateway.org/wp-content/uploads/2015/07/2LegStructure.png (accessed on 20 December 2024). These physical structural deficiencies will often lead to premature culling, removing affected sows from the breeding population and causing a loss of potential revenue. Research results indicate that increasing the average parity at removal by one-tenth could increase profit in the USA swine industry by USD 15 million [2]. Premature culling is often labeled and classified as locomotion disorders, which encompass a range of other problems, such as lameness, injuries to the sow and piglets, multiple syndromes, and general ambulatory unsoundness [4]. Due to the subjective nature of diagnostic techniques for assessing these problems, however, there have been numerous studies conducted to evaluate the correlation between sow locomotion, productivity, and mortality. The individual animal effects of locomotor dysfunctions affect the financial viability of intensively managed food animal production systems due to the detrimental effects on product production. These problems can also be particularly evident in [4] large commercial poultry meat [5] and dairy milk product [6] production, where animals are housed in confinement buildings. As with all food-producing animal systems, there are “downstream” effects of locomotor dysfunctions on the sustainability of these enterprises from an economic viability perspective [7].

### 1.2. Association Between Sow Locomotion and Mortality

The results of many studies indicate a close association between sow locomotion and mortality rates. Sow mortalities attributed to locomotion problems vary considerably. Estimates in studies are these mortalities range from 9% [8] to 23% [9]. It was found that lameness or foot lesions accounted for 8.6% of sow culling from breeding herds [10]. Similar results were reported with locomotion problems being responsible for 11% and 13% of sow culling during the early production stages [11]. In some of the initial studies, there was a focus on sow locomotion and motility, and there were similar findings with specific physical structure indicators that could be used to predict the risk of locomotion disorder occurrences [12]. The results from more recent studies are consistent in that sow longevity is an important animal welfare and economic concern for commercial swine breeders [3,13,14]. Locomotor dysfunctions, therefore, can significantly reduce efficiency in pork production enterprises due to increased mortality and other production-related inefficiencies associated with locomotor problems [15,16].

### 1.3. Subjectivity of Structural Appraisal in Sows

Visual evaluation of the structure, frame, and gait of sows can provide important information on the likelihood of an individual to have leg structural problems. Evaluations are often conducted by trained farm staff, and these manual inspections are considered standard practice in pork production enterprises. The inherent subjectivity in these assessments, however, has historically been a point of contention. The importance of reliable measurements for leg structural integrity was evident, with as many as 25% of females being culled because of issues with their feet and legs [7]. Furthermore, farm staff experience had effects on the consistency and repeatability of the scores for sow leg structural integrity [17,18]. Similar findings were subsequently reported, confirming the results of these studies [19,20].

The current objective measurement procedures for evaluating sow and gilt leg integrity need to yield data that are more quantitative. There are efforts to develop an objective measurement method of joint angles for knee, hock, front, and rear pasterns and a rear stance position in swine using digital imaging technology and to assess the repeatability of the objective measurement process [21]. Based on the results from the intraclass correlation coefficient analyses, the repeatability of the objective method used in this study to evaluate feet and leg structural soundness ranged from 0.552 to 0.879. It was concluded that an objective feet and leg structural soundness trait measurement could be implemented as an alternative to subjective methods because of the repeatability of determinations and the accuracy of joint evaluations.

The application of computer vision algorithms for evaluating images and video recordings of sows/gilts provides an unbiased and objective approach to assessing various indicators of foot and leg integrity. The results from a recent study in which computer vision models were developed to identify ten key body landmarks of pigs from their side profile images and two from their rear profile images resulted in a mean average precision (mAP) of 0.94 across all areas of the body that were evaluated [22]. Trigonometric formulae were developed to calculate the hock and knee angles from these body landmarks. These automated angle measurements were validated with comparisons to manual measurements. There was an average root mean square error (RMSE) of 4.13 deg and correlation coefficients (average r^2^ = 0.84) different from zero, confirming the consistency of the data when there were evaluations using the Bland–Altman procedure. This methodology, therefore, provided a reliable method for obtaining precise leg angle measurements, which can be valuable for refining gilt replacement criteria and ultimately enhancing sow breeding programs.

## 2. Body Weight Assessment of Pigs at Time of Marketing

Efficacious and efficient determinations of pig body weight is of great value in commercial swine production systems. The precise estimation of body weight influences economic profitability margins for swine producers and serves as an important indicator of health. For example, trends of decreasing body weight can be a potential indicator of disease and illness in food-producing animals. Detection can be enhanced using precision technologies [23]. Body weight is associated with dietary nutrient intake which, in turn, has biological implications for reproductive performance [24]. When body weight is routinely monitored, data can be plotted as growth curves, which is a useful production approach for evaluating feed efficiency utilization and pig growth in pork production enterprises. For these reasons, the accurate and precise estimation of animal body weight is of multifaceted significance and is particularly important at nearly every stage of production in pork-producing enterprises, but especially when determining optimal times for marketing pigs.

### 2.1. Current Techniques for Estimating Pig Body Weight

The approaches used in commercial pork production enterprises for estimating pig body weight are a direct measurement, using a scale, and visual estimates. The utilization of accurately calibrated scales will result in the collection of accurate body weight data, but this is labor-intensive and requires multiple individuals to effectively and efficiently collect data, which can result in injury and/or stress, leading to welfare issues for both pigs and those conducting the weighing task. Furthermore, a considerable time investment is required to obtain individual pig body weights using a scale, where employees could be conducting other duties instead [25]. The direct measurement technique, therefore, is impractical for large commercial pork production enterprises. The current alternative, using visual appraisal, has many other shortfalls. Visual estimations of pig body weight are not only highly subjective but also lack consistency, with accuracy depending on the experience of the farm staff [17].

### 2.2. Correlation Between Values for Pig Body Weight and Biometric Determinations

Historically, in numerous studies, there have been investigations of the relationship between pig body weight and quantifiable biometrics (such as heart girth, length, and height), with the consistent reporting of close correlations between body weight and these biometric variables. Three techniques have been compared for obtaining pig body biometric data: tape measure/caliper, livestock scales, and projections from 2D images of pigs [26]. The main findings were that calipers and measuring tapes were the most effective methods for estimating pig body weight. There are also close correlations between values for body weight and various biometric values such as height, length, width, flank, and girth, leading to the conclusion that the combination of biometric data can be used as independent variables to predict body weight [27]. Interestingly, the significant limitations reported for the utilization of biometric data in these studies were the same as those previously described when there were pig body weight determinations using scales: the need to immobilize the animals, inherent human measurement errors, subjectivity leading to non-reliable data, and time constraints. Considering the large number of pigs housed in present-day pork production enterprises, the manual collection of biometric data is not a feasible alternative to directly measuring pig body weight using scales.

### 2.3. Automated Electronic Weighing Systems

An emerging alternative to manual weighing is the use of automated weighing systems. While initially expensive, these systems are a viable alternative to manual weighing when combined with electronic identification tags, enabling individual animal tracking and automated data management. Without such tags, the benefits of automation are reduced. The initial findings were that a single employee could weigh as many as 100 pigs per hour by allowing pigs to pass through automated gates that led to the automatic collection of pig body weights [28]. There was a subsequent report that the automated approach was reliable with greater repeatability compared to manual weighing methods [29].

While both automated weighing systems and computer vision technologies offer promising alternatives to manual weighing in pig production, each alternative has unique implementation considerations. Automated weighing systems, while potentially resulting in less initial retrofitting costs than previously considered, require specific infrastructure modifications. Computer vision systems, even if there is reduced weight estimation accuracy and precision as compared with direct weighing using a scale, have advantages in terms of continuous, non-contact monitoring and the potential to collect additional data on animal behavior and body condition. Further research is needed to optimize the cost-effectiveness and practical application of both technologies, particularly in addressing challenges such as data interpretation for actionable management decisions and minimizing RFID tag loss when there is the utilization of automated weighing systems.

## 3. Computer Vision Techniques in Pig Assessment

Computer vision approaches are a promising solution for the objective collection of data, with there being the potential for automating the evaluation of feet and leg structural soundness and predicting pig body weight. The use of these technologies will enable continuous, noninvasive monitoring, providing consistent and accurate assessments that are important for effective pork production management. By automating the evaluation of key metrics such as structural soundness and body weight, the utilization of computer vision systems will lead to a reduction in the reliance for subjective visual assessments, thereby improving the reliability of the data used in decision-making processes. The application of computer vision, however, is not without challenges and requires a concerted research effort to fully realize its potential. In subsequent sections of this review article, the methods and application of computer vision techniques in making assessments in pork production enterprises are explored, focusing on the importance of enhancing the precision and efficiency of monitoring systems.

### 3.1. Introduction to Neural Networks

Neural networks are computational models that draw inspiration from the architecture and function of the human brain, enabling the processing of information and pattern recognition [30]. When well-trained neural networks are versatile, applications range from simple linear regression to categorizing animal behavior recognition. Details regarding what neural networks can be evaluated via information provided at this link: https://www.ibm.com/think/topics/neural-networks (accessed on 20 December 2024). The utilization of neural networks to detect and predict intricate patterns, particularly in complex data sources such as images, results in well-trained neural networks with great potential in various fields, including computer vision. By leveraging these capabilities, neural networks can break down images into fundamental components and progressively build to more complex representations, such as shapes and objects.

Neural networks consist of layers of interconnected nodes, referred to as neurons, that perform mathematical calculations. In computer vision applications, data in the form of images are passed through these layers and undergo a series of mathematical transformations. Each neuron applies a weighted sum to the inputs, which produces a “signal” that represents the neuron’s output at this stage. This signal is then passed through a nonlinear activation function, with there being the resulting decision of whether the signal should continue to propagate through the network or be deactivated. Signals that are deactivated are zeroed out from influencing subsequent layers of the final output. This selective process enables the network to learn from important features that activate the function while ignoring features that do not, enabling the network to learn complex patterns in the data. Training a neural network involves minor adjustments to the weights of the connections to minimize the error between the predicted output and the actual output, a process known as backpropagation. Ultimately, the final output of the network is a prediction or classification result that aligns with the data for which the network was trained.

### 3.2. Object Detection and Feature Extraction in Computer Vision

Computer vision techniques can be utilized to extract important visual features from images and videos. This field initially gained traction with the Deformable Parts Model (DPM), a pioneering object detection algorithm that relies on a structured support vector machine (SVM) rather than a neural network. The DPM represents objects as a collection of parts, each with specific geometric relationships, and uses a “sliding window” approach to scan the image and detect potential objects based on the alignment of these parts [31]. As the need for more efficient and faster object detection grew, the region-based convolutional neural network (R-CNN) was developed. The R-CNN improved upon the DPM by strategically assessing the “interesting” regions of an image instead of evaluating every possible region [32]. Although the R-CNN was an improvement compared to the DPM, the speed was inadequate for real-time applications. Subsequently, the “You Only Look Once” (YOLO) algorithm emerged as a more practical solution.

### 3.3. Advent of the “You Only Look Once” (YOLO) Algorithm

The development of the YOLO algorithm marked an advancement in object detection due to the time efficiency of detection and real-time capabilities for detection [33]. Both YOLO and the R-CNN are object detection models. These are designed to locate and classify objects, such as pigs or their body parts, within images and videos. The YOLO algorithm has become a central feature when utilizing computer vision approaches, with uses ranging from agricultural to facial recognition. The YOLO model has three main components: the backbone, neck, and head. The backbone is responsible for extracting essential features from the input image, the neck connects these features into a feature pyramid that enhances the network’s capacity to detect objects at different scales, and the head produces the final output, including the bounding boxes and class predictions.

What sets YOLO apart is “single-shot” detection capacities, which allows for evaluating the entire image in one pass, predicting both the objects that are present and their locations. This structure enables rapid object detection, resulting in YOLO being a more effective and efficient option as compared with previously utilized methods such as the R-CNN and DPM.

The application of the YOLO algorithm in pork production systems provides an important opportunity to advance precision agriculture. The use of this algorithm enables the precise collection of visual data, facilitating the objective and automated assessment of various aspects of pig anatomy. This capability has the potential to revolutionize animal husbandry by allowing producers to optimize feeding strategies, improve welfare conditions, and enhance productivity. By leveraging computer vision models such as YOLO, producers can make more informed decisions, ultimately increasing the efficiency and efficacy of pork production systems.

### 3.4. Localization in Computer Vision

With the utilization of YOLO, there is more precise localization, which leads to greater precision for the spatial location of objects within an image through boundary box and centroid coordinates. The utilization of this process begins with overlaying the image with randomly sized anchor boxes and the calculation of the Intersection of Union (IoU) for each anchor box against the “ground truth” (i.e., determined from data collected manually) annotation [33]. This allows for the determination of the confidence of each anchor box containing a class object. The utilization of this intricate procedure leads to the assurance that only the most statistically reliable boundary boxes are predicted, which leads to enhancing the accuracy of a trained YOLO model.

### 3.5. “Black Boxes” in Deep Learning

The utilization of CNNs has led to the efficacious estimating of pig body weight [34,35,36]. Convolutional neural networks (CNNs) have been proven to be highly effective in various applications, resulting in accurate predictions for conducting tasks involving complex visual data. A major barrier for the utilization of CNNs, however, is the “black box” nature, which refers to the lack of transparency in how the models make decisions. This opacity creates several challenges. First, it is difficult to pinpoint which specific features the model is using to make predictions, raising concerns about the reliability and consistency of these predictions across different datasets or conditions. For example, if a CNN model is trained on a particular dataset, it may rely on features that are not universally applicable, leading to potential inaccuracies when applied to a different dataset.

Furthermore, the inability to understand the internal workings of these models makes troubleshooting particularly challenging. When predictions are inaccurate or when the model fails to perform as expected, it can be difficult for scientists, clinicians, or producers to diagnose the root cause of the problem. This lack of interpretability can be a major barrier to the broader utilization of CNNs in practical applications, as end-users may be hesitant to trust a system they cannot fully understand or explain.

For scientists and researchers, the “black box” nature of CNNs limits the capacity to extract meaningful insights from the data, which is essential for advancing knowledge and refining models. To address these issues, there is a growing interest in developing more interpretable models or enhancing the transparency of existing CNNs using techniques like explainable AI (XAI). With these efforts, there is an attempt to bridge the gap between the useful predictive capabilities of CNNs and the need for definitive, understandable decision-making processes in fields like animal husbandry and precision agriculture.

### 3.6. Metrics of Assessing Computer Vision Models

The performance of computer vision models, particularly in object detection and classification tasks, is commonly evaluated using metrics that provide insight into accuracy and reliability. Important metrics include mean average precision (mAP) [37], Intersection of Union (IoU) [38], precision, and recall [39]. Mean average precision is a widely subscribed-to metric that combines precision (i.e., the ratio of true positive predictions to all positive predictions) and recall (i.e., the ratio of true positive predictions to all actual positives across various thresholds). This metric provides for an aggregated measure of a model’s capacity to correctly identify objects, reflecting its overall performance when there are varying extenuating circumstances. Intersection of Union (IoU) assesses the accuracy of object localization by measuring the overlap between predicted bounding boxes and the actual bounding boxes where data were collected manually, which are often manually labeled. The IoU ranges from 0 to 1, where a larger IoU indicates more precise localization, making it particularly valuable in applications where accuracy in locating objects within an image is essential.

In traditional statistics, precision evaluations are a simpler determination of a model’s predictive capabilities by indicating the likelihood that a positive prediction is correct. It, however, does not account for the model’s capacity to detect all real positives (recall) or accuracy in localization (IoU).

Ultimately, mAP and IoU together offer a nuanced assessment tailored to the unique challenges of computer vision tasks such as object detection. While mAP reflects the model’s overall detection performance, IoU provides a direct measure of localization accuracy. These metrics, alongside precision and recall, provide a comprehensive toolkit for quantitatively assessing the efficacy of computer vision models.

## 4. Application of Computer Vision in Pig Assessment

### 4.1. Estimating Pig Body Weight

Computer vision has emerged as a promising alternative for overcoming the limitations of conventional pig body weight estimation. Biometric methods for body weight estimation in pigs have been determined to be accurate and reliable [40]. Computer vision systems can be utilized to determine biometric measurements with precision, making these systems candidates for integration. These systems can be specifically modified for use in pork production enterprises and are noninvasive, automated, and allow for the collection of objective data for determining biologically important features of food-producing animals.

#### 4.1.1. Traditional Image Processing Techniques for Pig Body Weight

Traditional image processing techniques differ significantly from deep learning-based methods, like YOLO and RCNN. These are designed to use manually crafted algorithms and features to process images. By design, these are suitable for simple tasks (such as thresholding) but are not very useful with complex “real-world” scenarios (thresholding in varying lighting). In an early study, a traditional computer vision technique was utilized to evaluate the dorsal pig surface, and from the data collected, there was the estimation of pig body weight with an accuracy of 5% [41]. Similarly, when contrasting lighting conditions were evaluated to estimate the dorsal surface area of pigs, there were predictions of pig body weight within an accuracy of 0.9 kg [42]. Utilization of an eclipse fitting model, and the Hough transform image extraction technique, resulted in an estimated pig body weight to an accuracy of 96.2% with an average error of 1.23 kg [43]. Even with these advancements, one significant limitation of these 2D image processing techniques is the lack of a capacity to obtain a comprehensive variety of relevant biological features. For example, in these previous studies, the dorsal images of pigs could be evaluated and utilized for effectively determining pig width and back length, but this approach is not effective for determining pig girth or flank measurements. Furthermore, with the use of these techniques, there is the utilization of Euclidian distances, which are not effective at determining geodesic features such as pig girth.

#### 4.1.2. Three-Dimensional Computer Vision Techniques for Pig Body Weight

While 2D computer vision is important in the detection of live animals, the advent of 3D computer vision was a transformative advancement. This technology enables the capture of 3D geodesic data, which include important measurements such as the girth of an animal, ultimately expanding the scope of what is possible with the precision assessment of body weight in food-producing animals. With the use of depth imaging procedures, there is the utilization of 3D data, a compelling alternative to relying solely on 2D images. One pioneering utilization of depth imaging for swine-related research included mounting an RGB depth camera on the ceiling of a pig housing facility. In this study [44], an RGB-D computer vision system was developed for predicting the body weight of non-restrained pigs using top-view RGB and depth images. For 38 days, images of eight pigs were recorded via video daily using an Intel RealSense D435 camera for 3 min at six frames per second, while ground truth weights were determined directly using a scale. Pigs were of crossbred Yorkshire and Large White breeding and were 5 weeks of age at the initiation of the experiment. On average, the pigs weighed 23.5 kg (SD = 7.6 kg) at the start of the experiment and 46.7 kg (SD = 8.7 kg) at experimental cessation. Morphological features, such as length, width, and height, were extracted using Python’s OpenCV library. By conducting quality control evaluations, there was the removal of frames with motion blur, distorted shapes, or non-standard positions. Linear mixed models were used to make predictions, achieving coefficients of determination ranging from 0.49 to 0.98, highlighting the robustness and potential for widespread application in pork production enterprises.

In a similar study, there was the validation of the use of depth images for predicting pig body weight [45]. A total of 772 depth images and corresponding mass measurements were collected from 234 pigs in a grow-finish pork production facility of pigs of Landrace, Duroc, and Yorkshire breeding. Within this group, there was an equal number of barrows and gilts. On average, piglets weighed 27 kg (SD = 4.4 kg) at the start and 40 kg (SD = 6.5) at the end of the experiment. Body weight was calculated from these depth images collected from a Kinect sensor (Microsoft, Milipitas, CA, USA) and mounted on the wall above the animal scale. Both color and depth images were acquired at approximately 1 s intervals. Metrics were collected using a program developed in MATLAB software (version R2015b). Using this software, a system of linear equations was developed to predict the weight from the volume. The global equation was determined to be R^2^ = 0.9905. These findings indicate that depth sensors can provide an accurate and scalable solution to continuous body weight estimation.

There was another study where there was use of photogrammetry and artificial neural networks to estimate the body weight of Holstein cattle [46]. Body dimensions (wither height, hip height, body length, and hip width) were captured using Canon EOS 400D cameras (Canon, Oita, Japan) that were synchronized and calibrated to obtain 3D data. From the resulting data, metrics on body dimensions were collected. This dataset was divided into test and training subsets, with the most precise performing ANN having reliable accuracy in predicting pig body weight. The correlation coefficient was 0.995 when compared to weights obtained manually. These results indicate that photogrammetrically derived body metrics can be a reliable method for predicting pig body weight.

The use of 3D point clouds allows for three-dimensional representations of the animal, capturing spatial information that goes beyond two-dimensional imaging. Early research explored the potential of 3D point cloud technology for accurate pig body weight estimations. In a recent study [47], a hybrid approach was utilized, combining statistical filtering and DBSCAN clustering for denoising point clouds. This technique mitigated bias and improved feature extraction. The model incorporated pig dorsal body area parameters and a CNN, achieving a mean absolute error of 12.45 kg and a mean absolute percentage error of 5.36%. In a recent study [48], a 3D deep learning approach on point clouds collected in a pen environment called PointNet was utilized. PointNet is a model used to process point cloud data, which consist of points in 3D space, representing the shape and structure of objects. With the utilization of this model, there was a coefficient of determination of 0.94 and a root mean squared error of 6.88 kg. Comparing the PointNet model results to that of a volume-based method, there was an improved accuracy (R^2^ = 0.94 compared with 0.75). Concurrently, ref. [49] there was the development of a non-contact model using point cloud data from the dorsal evaluation of the pig’s anatomy, with there being an absolute error of 11.552 kg and a relative error of 4.812%. The findings in these studies highlight the potential of 3D point cloud technology to provide accurate and noninvasive techniques for estimating pig body weight. Challenges with point cloud technology remain, such as the computational requirements for processing large point cloud datasets, interference from environmental factors such as dust and airborne particles, and the need for standardization across different sensor systems. Nonetheless, advancements in hardware and software have promise to improve the potential of 3D point cloud technology in food-producing animal management.

While the findings from these results are promising, it is essential to assess the robustness of models when analyzing datasets that have not been previously evaluated using this model. Image data can vary in terms of environmental conditions, lighting, position of the animal, size, color, and age. For example, models trained on specific breeds or age groups will likely not be effective when evaluating animals of other ages, e.g., as in [20], because models are trained on specific key anatomical features. Depth data accuracy is also affected by hardware variability, camera angles, lighting conditions, and housing environment. These changes can potentially limit the robustness of a depth estimation model in less controlled settings. Depth imaging technology is highly susceptible to “noise” resulting from dust in the air, ambient lighting, and the occlusion of objects, impacting the precision of 3D measurements collected from these types of sensors. To address these challenges, improvements include the collection of more diverse datasets across different enterprises, breeds, age groups, and other conditions to improve the breadth of model utilization. Incorporating advanced filtering techniques or using more sophisticated sensors like a multi-camera array can help reduce noise and increase the resolution of captured data. The integration of CNN’s successful 2D image tasks, with depth sensors to capture complex 3D data patterns, has recently been utilized to conduct studies with food-producing animals. The results from recent studies indicate the practical efficacy of these approaches. For example, in [50], the utilization of depth images and CNNs, enhanced with transfer learning and model ensembling, to estimate body condition scores in dairy cows, resulted in a relatively precise accuracy (82% within 0.25 BCS units; 97% within 0.50 units). Similarly, an automated cow body condition scoring system using multiple 3D cameras has been developed, along with the training of independent CNN models and combining the estimations through ensemble modeling for significantly improved accuracy [51]. These findings suggest that similar techniques can be effectively applied to other food-producing animals, including swine.

### 4.2. Scoring of Feet and Leg Structural Soundness

Before the advent of deep learning technology, there was the evaluation of objective and automated techniques to quantify animal leg soundness [52,53,54,55]. These studies were initially conducted with horses and cattle but were the foundational information for a similar assessment of pigs. Subsequently, pig-related research was conducted where there were evaluations of sow feet and leg soundness traits using video recordings [56]. There, however, was a small correlation between values for manually measured traits and those obtained using automated techniques, which is indicative of the need for more precise measurement approaches. There were also computer vision techniques evaluated to assess the feet and leg structural soundness of pigs [57]. The results from these studies are indicative of the fact that computer vision has potential to assess intricate details, such as joint angles, stride lengths, and gait patterns, ultimately allowing for a more precise assessment opportunity for physical traits, such as feet and leg soundness.

### 4.3. Challenges in Adopting Machine Learning Technologies

While many of the previously discussed technologies can be used without internet connectivity, the development, testing, and refinement of machine learning models involve significant computational complexity and large dataset management requirements. Training datasets for models can range from a few gigabytes to hundreds of gigabytes, as demonstrated by version 3.5 of the large language model (LLM) ChatGPT (OpenAI, San Francisco, CA, USA), where there was the utilization of 570 GB of training data [58]. Beyond connectivity, the computational infrastructure needed to train and utilize these models requires access to specialized hardware, such as GPUs or TPUs, and robust storage systems that are inaccessible on most rural farms. This makes deployment in these technologies challenging unless cloud-based solutions or edge computing devices are leveraged.

Edge computing, a technology that processes data locally on devices rather than relying on centralized infrastructure, offers a promising solution by enabling real-time data processing. This reduces dependency on high-speed internet and is particularly advantageous in rural settings, where local devices can perform important computations and continue to function independently from internet connectivity. By integrating cloud computing with local deployments, farmers can benefit from a hybrid system that enhances feasibility and functionality in rural farms. For example, the results from one study [59] are indicative of how cloud computing can be integrated with remote sensing technologies to optimize farming practices. This system was tested in rural agricultural settings and addressed network and resource limitations to enhance crop yield and resource efficiency. This integrated system highlights the feasibility of combining cloud-based and localized systems to improve the adoption of machine learning in resource-constrained settings.

Furthermore, adopting ML technologies requires technical expertise in data management, model training, and integration into farm workflows. Many farmers lack access to training or resources to effectively use this software and hardware-based tools, creating a barrier to machine learning adoption on the farm. Lightweight models, such as MobileNet [60], or techniques such as model pruning, are specifically designed for resource-constrained settings. These approaches make ML tools more accessible and practical for farmers in rural areas. For example, MobileNet employs depthwise separable convolutions to reduce computational complexity, resulting in less power consumption and faster inference times when compared to traditional convolutional neural networks. The results indicated that the quantized version of SSD-MobileNet-v2 has an inference time of ~68.96 ms with a COCO mAP of 60.99, while the default SSD model requires greater than 120 ms per inference with a similar mAP score [58]. This increase in speed makes lightweight models particularly advantageous for deployment in rural settings where computational resources are limited. Even when edge computing devices are used to process the data locally, there are still challenges, such as maintenance, software updates, and troubleshooting of the devices. These problems require skilled personnel, which adds to operational costs. Furthermore, ethical considerations such as data privacy and security remain pertinent, particularly when transmitting sensitive food animal production unit-derived data to centralized data centers.

High-speed internet connectivity is necessary for transmitting large datasets to data centers for model training and integration. Many food animal producers in rural areas in the USA still lack access to advanced broadband services (100 mb/s download, 20 mb/s upload) [61,62]. This lack of connectivity limits the adoption of technologies such as centralized cloud-based herd management and video-assisted evaluations of food-producing animals, which require robust broadband for real-time analysis. While these techniques have potential, the reliance on stable network connections creates barriers in rural areas without sufficient infrastructure. To address this, offline deployment strategies, such as preloading essential data or enabling models to inference offline [63], can ensure uninterrupted functionality. Lightweight ML models, such as MobileNet, further enhance this by allowing for efficient computations on resource-constrained devices. When combined with hybrid approaches that integrate edge and cloud computing, localized real-time processing can be achieved while still periodically syncing with the cloud for updates and more precise analytics. These layered solutions integrate the benefits of cloud-based systems, lightweight models, and offline strategies, making these effective for overcoming connectivity challenges in rural settings.

## 5. Conclusions

The evolution of the pork production industry has been marked by continuous changes and a quest for precision, objectivity, and optimization. While traditional techniques have served the industry for centuries, the limitations of manual techniques, particularly for pig body weight estimation and feet and leg structural soundness, have become evident. Computer vision approaches allow for objective, automated, and noninvasive techniques to be utilized for evaluating pigs. From trained deep learning detection models to complex algorithms for 3D feature extraction, the advancements in computer vision procedures are going to rapidly change the way producers assess pigs in pork production systems. As the industry continues to evolve, there is no doubt that computer vision approaches will be pivotal in shaping the future of pork production.

There is an opportunity to integrate computer vision technologies with walk-over-weighing technologies for evaluating structural characteristics (e.g., structural soundness integrity) and sow body weight changes in pork production systems. The further refinement of precision technologies and access to adequate internet connectivity will enhance technology implementation in pork production, similar to advancements in other food animal (e.g., dairy) and food grain production systems.

## Data Availability

No new data were created or analyzed in this study.
